# The complete mitochondrial genome of *Microphysogobioelongatus* (Teleostei, Cyprinidae) and its phylogenetic implications

**DOI:** 10.3897/zookeys.1061.70176

**Published:** 2021-10-01

**Authors:** Renyi Zhang, Qian Tang*, Lei Deng

**Affiliations:** 1 Key Laboratory of National Forestry and Grassland Administration on Biodiversity Conservation in Karst Mountainous Areas of Southwestern China, School of Life Sciences, Guizhou Normal University, 550001, Guiyang, Guizhou, China Guizhou Normal University Guiyang China

**Keywords:** Gobioninae, mitogenome, paraphyly

## Abstract

Mitochondria are important organelles with independent genetic material of eukaryotic organisms. In this study, we sequenced and analyzed the complete mitogenome of a small cyprinid fish, *Microphysogobioelongatus* (Yao & Yang, 1977). The mitogenome of *M.elongatus* is a typical circular molecule of 16,612 bp in length containing 13 protein-coding genes (PCGs), 22 transfer RNA genes, two ribosomal RNA genes, and a 930 bp control region. The base composition of the *M.elongatus* mitogenome is 30.8% A, 26.1% T, 16.7% G, and 26.4% C. All PCGs used the standard ATG start codon with the exception of *COI*. Six PCGs terminate with complete stop codons, whereas seven PCGs (*ND2*, *COII*, *ATPase 6*, *COIII*, *ND3*, *ND4*, and *Cyt b*) terminate with incomplete (T or TA) stop codons. All tRNA genes exhibited typical cloverleaf secondary structures with the exception of tRNA^Ser(AGY)^, for which the dihydrouridine arm forms a simple loop. The phylogenetic analysis divided the subfamily Gobioninae in three clades with relatively robust support, and that *Microphysogobio* is not a monophyletic group. The complete mitogenome of *M.elongatus* provides a valuable resource for future studies about molecular phylogeny and/or population genetics of *Microphysogobio*.

## Introduction

The genus *Microphysogobio* Mori, 1934, small gudgeons of the subfamily Gobioninae, was originally established by Mori (1934) for *M.hsinglungshanensis* Mori, 1934 (Sun et al. 2021). Currently, this genus comprises approximately 30 species that are widely distributed in East Asia, including China, Vietnam, Mongolia, Laos, and the Korean Peninsula (Jiang et al. 2012; Huang et al. 2016; Huang et al. 2017). The prominent feature of the lip papillae was considered a diagnostic character for defining the genus *Microphysogobio* and distinguishing it from other genera in the subfamily Gobioninae (Yue 1998). Molecular phylogenetic studies of the subfamily Gobioninae has confirmed the monophyletic nature of the Gobioninae (Tang et al. 2011; Zhao et al. 2016). However, the phylogenetic relationships of *Microphysogobio* and related genera have not been fully resolved, and it is a long-standing issue in the classification of Gobioninae.

The typical vertebrate mitogenome is approximately 15–18 kb in length, consisting of 13 protein-coding genes (PCGs), 22 transfer RNA (tRNA) genes, two ribosomal RNA (rRNA) genes, and one non-coding control (*D-loop*) region (Wolstenholme 1992; Boore 1999). Mitochondrial genomic DNA has the following characteristics: small size, multiple copies, maternal inheritance, conservative gene products, no introns, fast evolutionary rate, and rare recombination (Boore 1999; Xiao and Zhang 2000). Therefore, it is widely used in species identification, molecular evolution, and phylogenetic studies (Imoto et al. 2013; Sharma et al. 2020). Historically, several genes on the mitochondrial genome, such as *Cyt b* gene and *D-loop* (Wang et al. 2002; He and Chen 2006) were used to study the evolutionary relationships. More recently, with advances in sequencing technology and data analysis methods, information on fish mitogenomes has been accumulating in public databases (Miya and Nishida 2000; Miya et al. 2003; Saitoh et al. 2006; Yamanoue et al. 2007; Kim et al. 2009).

*Microphysogobioelongatus* (Yao & Yang, 1977) is a small, benthic, freshwater fish which is widely distributed in China (Yue 1998; Wang 2019). However, little is known regarding *M.elongatus*, with previous studies focusing on resources investigation and taxonomy (Li et al. 2012; Liu et al. 2013; Zhang et al. 2018). In this study, we sequenced, annotated, and characterized the complete mitochondrial genome of *M.elongatus*. Additionally, we reconstructed the mitogenomic phylogeny of Gobioninae, involving 103 species and subspecies based on 13 PCGs to confirm the taxonomic status of *M.elongatus* and its relationships within Gobioninae.

## Materials and methods

### Ethics statements

For field collection, no specific permissions are required for the collection of gobionine fishes from public areas. The field collections did not involve endangered or protected species, and the collection site is not a protected area.

### Sample collection and DNA extraction

Individuals of *M.elongatus* were collected from Jiangkou County, Guizhou Province, China (27°46'12"N, 108°46'56"E), in August 2019. The specimens were preserved in 95% ethanol and stored at –20 °C until DNA extraction. Genomic DNA was extracted using a standard high-salt method (Sambrook et al. 1989). The integrity of the genomic DNA was measured by 1% agarose gel electrophoresis, and the concentration and purity of DNA were determined using an Epoch 2 Microplate Spectrophotometer (Bio Tek Instruments, Inc., Vermont, USA).

### PCR amplification and sequencing

The entire mitogenome of *M.elongatus* was amplified in overlapping PCR fragments by 14 primer pairs designed from the mitogenome of *M.kiatingensis* (GenBank accession number NC_037402) by Primer Premier v. 5.0 software (Lalitha 2000). The primers used in this study are provided in Suppl. material 1: Table S1. Each PCR reaction was carried out in 35 μL total volume, containing 17.5 μL of 2×Taq Plus MasterMix (CoWin Biosciences, Beijing, China), 1 µL of each primer (10 µM) and 1.0 μL of template DNA (100 ng). The PCR reactions were performed under the following conditions: an initial pre-denaturation at 95 °C for 5 min, 35 cycles of 95 °C for 30 s, 42–55 °C for 30 s, 72 °C for 1–2 min, and a final extension at 72 °C for 10 min. Amplification products were fractionated by electrophoresis through 1% agarose gels. The lengths of fragments were determined by comparison with the DL2000 DNA marker (TaKaRa, Japan). The PCR products were sequenced by ABI PRISM 3730 (Sangon Biotech. Co., Ltd, China).

### Mitogenome annotation and sequence analysis

The mitogenome was initially assembled by the SeqMan software of DNAStar (DNASTAR Inc., Madison, WI, USA), then manually proofread based on sequencing peak figures. The assembled mitogenome sequence was subsequently annotated using MitoAnnotator on the MitoFish homepage (Iwasaki et al. 2013). All tRNA genes were identified with tRNAscan-SE search server (Lowe and Chan 2016) and MITOS WebServer (Bernt et al. 2013). The base composition, codon usage, and relative synonymous codon usage (RSCU) of all PCGs were calculated using MEGA v. 6.0 (Tamura et al. 2013). Strand asymmetry was calculated using the following formulae: AT-skew = (A – T) / (A + T) and GC-skew = (G – C) / (G + C) (Perna and Kocher 1995).

### Phylogenetic analysis

For phylogenetic analysis, 103 gobionine fishes were downloaded from GenBank. Additionally, *Acheilognathusomeiensis* (NC_037404.1), *Rhodeusocellatus* (NC_011211.1), and *R.sinensis* (NC_022721.1) were used as outgroups. Species used in the analysis are listed in Suppl. material 2: Table S2. The shared 13 concatenated protein-coding genes (PCGs) were extracted and recombined to construct a matrix using PhyloSuite v. 1.1.16 (Zhang et al. 2020). The 13 PCGs were aligned separately using MAFFT v. 7.313 (Katoh and Standley 2013) and concatenated. The optimal partition strategy and nucleotide sequence substitution model of each partition were estimated by PartitionFinder v. 2.1.1 (Lanfear et al. 2017) with the Corrected Akaike information criterion (AICc) algorithm under a greedy search. A Bayesian inference (BI) analysis was performed using MrBayes v. 3.2.6 (Ronquist et al. 2012) with the models determined by PartitionFinder. Two independent runs of four Markov Chain Monte Carlo (MCMC) chains (one cold chain and three heated chains) were performed for two million generations sampling every 100 generations. The first 25% of the generations were discarded as burn-in and a 50% majority rule consensus tree was constructed. A maximum likelihood (ML) analysis was performed using IQ-TREE v. 1.6.8 (Nguyen et al. 2015) with 10,000 bootstrap replicates using the ultrafast bootstrapping algorithm (Minh et al. 2013). All software were integrated into PhyloSuite v. 1.1.16 (Zhang et al. 2020). The phylogenetic trees were visualized using FigTree v. 1.4.2 (http://tree.bio.ed.ac.uk/software/figtree/).

## Results and discussion

### Genome organization and nucleotide composition

**Figure 1.**
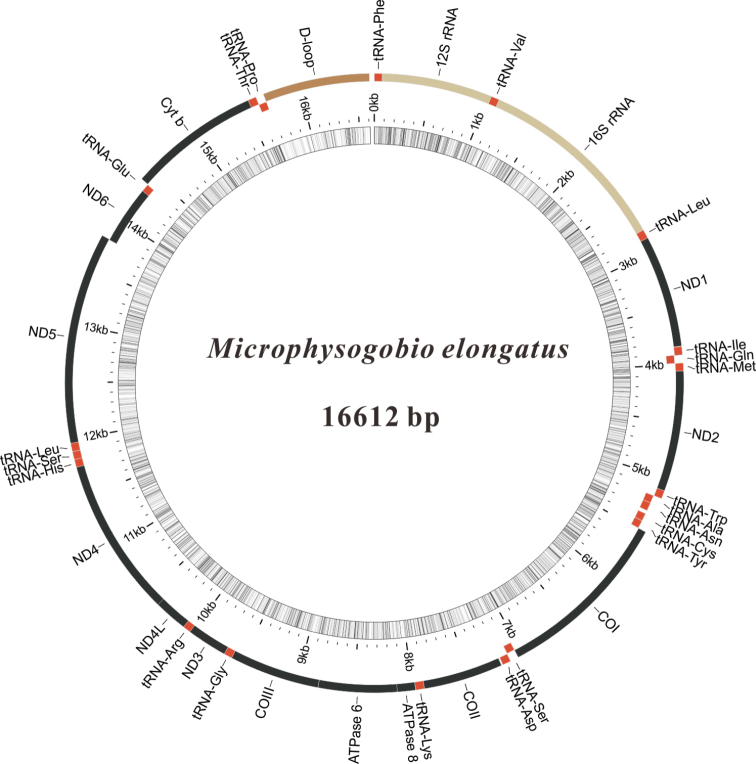
Circular map of the M.elongatus mitogenome.

The complete mitochondrial genome of *M.elongatus* was first reported and analyzed in this study. The full length of the *M.elongatus* mitochondrial genome sequence had 16,612 bp. The complete mitochondrial genome of *M.elongatus* was annotated and submitted to GenBank (GenBank accession number MN832777). It consisted of 13 PCGs, 22 tRNA genes, two rRNA genes, and one control region (Fig. 1; Table 1). All mitochondrial genes were encoded on the heavy strand (H strand), except the *ND6* gene and eight tRNAs (Table 1). The arrangement and content of these genes were conserved and typical of *Microphysogobio* mitochondrial genomes (Hwang et al. 2014; Lin et al. 2014; Cheng et al. 2015). The *M.elongatus* mitogenome contained a total of 21 bp overlapping regions which were in six pairs of neighboring genes, ranging from 1 to 7 bp in length. The longest overlapping region (7 bp) was located between *ATP8* and *ATP6*, *ND4L* and *ND4*. A total of 65 bp intergenic nucleotides (IGN) were dispersed in 13 locations, ranging from 1 to 31 bp in length (Table 1). The longest intergenic spacer was located between tRNA^Asn^ and RNA^Cys^. These overlapping and intergenic regions are very common in fish mitochondrial genomes (Zhang and Wang 2018; Wang et al. 2020).

**Table 1.** Mitochondrial genome organization of *Microphysogobioelongatus*.

Gene	Strand	Position		Length (bp)	Intergenic nucleotide	Anticodon	Codon	
		From	To				Start	Stop
*tRNA-Phe*	H	1	69	69	0	GAA		
*12S rRNA*	H	70	1029	960	0			
*tRNA-Val*	H	1030	1101	72	0	TAC		
*16S rRNA*	H	1102	2793	1692	0			
*tRNA-Leu (UUR)*	H	2794	2869	76	1	TAA		
*ND1*	H	2871	3845	975	4		ATG	TAG
*tRNA-Ile*	H	3850	3921	72	-2	GAT		
*tRNA-Gln*	L	3920	3990	71	1	TTG		
*tRNA-Met*	H	3992	4060	69	0	CAT		
*ND2*	H	4061	5106	1046	0		ATG	TA-
*tRNA-Trp*	H	5107	5177	71	2	TCA		
*tRNA-Ala*	L	5180	5248	69	1	TGC		
*tRNA-Asn*	L	5250	5322	73	31	GTT		
*tRNA-Cys*	L	5354	5421	68	2	GCA		
*tRNA-Tyr*	L	5424	5493	70	1	GTA		
*COI*	H	5495	7045	1551	0		GTG	TAA
*tRNA-Ser (UCN)*	L	7046	7116	71	3	TGA		
*tRNA-Asp*	H	7120	7191	72	13	GTC		
*COII*	H	7205	7895	691	0		ATG	T—
*tRNA-Lys*	H	7896	7971	76	1	TTT		
*ATPase 8*	H	7973	8137	165	-7		ATG	TAA
*ATPase 6*	H	8131	8813	683	0		ATG	TA-
*COIII*	H	8814	9597	784	0		ATG	T—
*tRNA-Gly*	H	9598	9669	72	0	TCC		
*ND3*	H	9670	10019	350	0		ATG	TA-
*tRNA-Arg*	H	10020	10088	69	0	TCG		
*ND4L*	H	10089	10385	297	-7		ATG	TAA
*ND4*	H	10379	11760	1381	0		ATG	TA-
*tRNA-His*	H	11761	11829	69	0	GTG		
*tRNA-Ser (AGY)*	H	11830	11898	69	1	GCT		
*tRNA-Leu (CUN)*	H	11900	11972	73	0	TAG		
*ND5*	H	11973	13808	1836	-4		ATG	TAG
*ND6*	L	13805	14326	522	0		ATG	TAG
*tRNA-Glu*	L	14327	14395	69	5	TTC		
*Cyt b*	H	14401	15541	1141	0		ATG	T—
*tRNA-Thr*	H	15542	15613	72	-1	TGT		
*tRNA-Pro*	L	15613	15682	70	0	TGG		
*D-loop*	H	15683	16612	930	0			

The nucleotide composition of the *M.elongatus* mitogenome was as follows: 30.8% A, 26.1% T, 16.7% G, and 26.4% C, and were slightly (56.9%) A+T rich (Table 2). In addition, the A+T contents of PCGs, rRNAs, and tRNAs were also slightly A+T rich (Table 2). Compared to the entire mitogenome, the control region, known as an A+T rich region, contained the highest A+T content (68.1%) (Table 2). The skew statistics revealed a positive AT-skew and a negative GC-skew across the whole mitogenome (Table 2), indicating a bias toward As and Cs.

**Table 2.** Nucleotide composition of the *Microphysogobioelongatus* mitochondrial genome.

	Length(bp)	A%	T%	G%	C%	A+T%	AT-skew	GC-skew
Genome	16612	30.8	26.1	16.7	26.4	56.9	0.081	–0.226
PCGs	11423	28.7	28.2	16.2	26.9	56.9	0.009	–0.249
1^st^ codon position	3808	27.7	29.6	16.0	26.7	57.3	–0.032	–0.251
2^nd^ codon position	3808	30.1	27.5	14.5	27.9	57.6	0.045	–0.318
3^rd^ codon position	3807	28.3	27.4	18.2	26.1	55.7	0.016	–0.179
rRNA	2652	34.2	20.0	21.2	24.6	54.2	0.261	–0.073
tRNA	1562	28.4	26.9	23.5	21.2	55.3	0.028	0.052
*D-loop* region	930	34.2	33.9	13.3	18.6	68.1	0.005	-0.165

### Protein-coding genes and codon usage

The 13 PCGs were 11,423 bp in total length. The longest PCG was 1836 bp (*ND5*), and the shortest was 165 bp (*ATP8*) (Table 1). The average base composition of the 13 PCGs were as follows: 28.7% A, 28.2% T, 16.2% G, and 26.9% C (Table 2). All PCGs were initiated with the typical ATG codon except *COI* with GTG as its initiator codon. Six PCGs (*ND1*, *COI*, *ATPase 8*, *ND4L*, *ND5*, and *ND6*) terminated with a complete stop codon. The others terminated with an incomplete stop codon TA- or T—, which would be completed as TAA by post-transcriptional polyadenylation at the 3' end of the mRNA (Ojala et al. 1981).

The relative synonymous codon usage (RSCU) values of the 13 PCGs were analyzed and shown in Suppl. material 5: Fig. S5 and Suppl. material 3: Table S3. The total number of codons, excluding termination codons, in the 13 PCGs was 3808 (Suppl. material 3: Table S3). Among them, CUA, AUU, and UUA were most frequent. Seven codons (AAG, UCG, AGG, AGA, CGC, CGU, and GCG) were rarely represented. Furthermore, the three most frequent amino acids were Leu, Ser, and Ile (Suppl. material 6: Fig. S6).

### Transfer and ribosomal RNAs

The mitogenome of *M.elongatus* contains 22 tRNAs, which were interspersed across the circular genome, ranging from 68 bp (tRNA^Cys^) to 76 bp (tRNA^Leu (UUR)^ and tRNA^Lys^) in length (Table 1). The secondary structure of all tRNA sequences were predicted and the results showed they are capable of folding into typical cloverleaf secondary structures except for tRNA^Ser(AGY)^, in which the dihydrouridine (DHU) arm did not form a stable structure (Suppl. material 7: Fig. S7). This unique secondary structure has been commonly witnessed in many other fishes (Zhang and Wang 2018; Zhong et al. 2018). The average base composition of the tRNAs was 28.4% A, 26.9% T, 23.5% G, and 21.2% C (Table 2).

The 12S rRNA and 16S rRNA were the only two ribosomal genes in the mitogenome of *M.elongatus*. They were 960 bp and 1692 bp in length, respectively (Table 1). Similar to other fishes (Broughton et al. 2001; Zhang and Wang 2018), the 12S rRNA and 16S rRNA were located between tRNA^Phe^ and tRNA^Val^, and between tRNA^Val^ and tRNA^Leu (UUR)^, respectively (Table 1). Their average base composition was as follows: 34.2% A, 20.0% T, 21.2% G, and 24.6% C. The average A + T content of both rRNAs was 54.2% (Table 2). The lengths and A + T content of these two rRNAs were well within the ranges observed in other *Microphysogobio* mitogenomes (Lin et al. 2014; Hwang et al. 2014; Cheng et al. 2015).

### Mitochondrial control region

The mitochondrial control region (CR), or *D-loop*, is responsible for replication and transcription of the mitogenome (Boore 1999). The CR of *M.elongatus* was 930 bp in length and located between tRNA^Phe^ and tRNA^Pro^. Multiple homologous sequence alignment revealed three conserved structures (termination-associated sequence (TAS), central conserved sequence blocks (CSB-F, CSB-E, and CSB-D) and conserved sequence blocks (CSB-1, CSB-2, and CSB-3)) within the CR (Suppl. material 8: Fig. S8), as seen in most fish mitogenomes (Broughton et al. 2001; Zhang and Wang 2018).

### Mitochondrial phylogeny within Gobioninae

**Figure 2.**
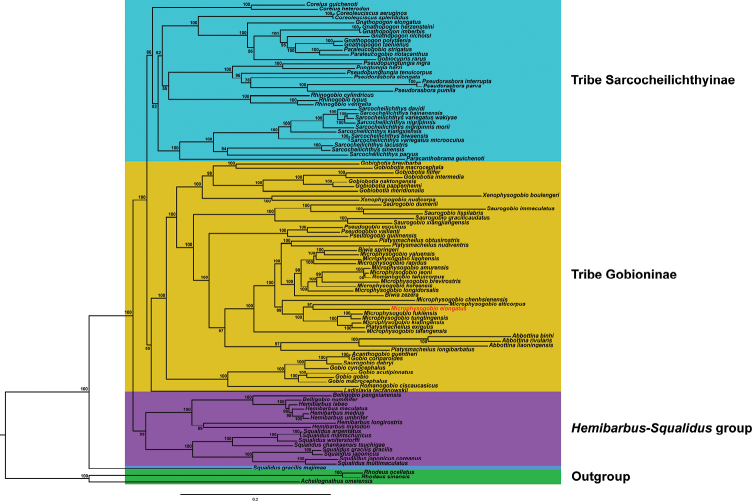
Phylogenetic relationships of Gobioninae based on complete mitochondrial genomes using maximum likelihood (ML) analyses. ML bootstrap values are shown at the nodes.

We reconstructed the phylogenetic tree of gobionine fishes based on the 13 concatenated protein-coding genes. The optimal partitioning scheme for the dataset and the best-fitting substitution model for each partition were provided in Suppl. material 4: Table S4. The trees resulting from the BI and ML analyses showed a consensus topology, and the only differences were the Bayesian posterior probabilities and ML bootstrap values (Fig. 2, Suppl. material 9: Fig. S9). The phylogenetic analysis revealed that Gobioninae could be separated into three clades (Tribe Sarcocheilichthyini, Tribe Gobionini and *Hemibarbus*-*Squalidus* group) with *Squalidusgracilismajimae* excluded (Fig. 2), which was consistent with previous phylogenetic studies (Tang et al. 2011; Zhao et al. 2016).

The *Hemibarbus*-*Squalidus* group includes *Belligobio*, *Hemibarbus*, and *Squalidus* (BS = 99%, PP = 100%). The *Hemibarbus*-*Squalidus* group was located at the basal position Gobioninae in the phylogenetic tree. This confirmed morphology-based hypothesis that *Hemibarbus* and *Belligobio* might represent the primitive group of Gobioninae (Bănărescu 1992). *Hemibarbus* and *Belligobio* were similar in morphological, and therefore, Bănărescu and Nalbant (1973) assigned *Belligobio* as a subgenus of *Hemibarbus*. The phylogenetic tree of Gobioninae subfamily based on single gene confirmed the close relationship of *Squalidus* to *Hemibarbus* (Yang et al. 2006; Liu et al. 2010; Tang et al. 2011). Nonetheless, the phylogenetic tree suggests that the classification of *S.g.majimae* should be further revised.

The tribe Gobioninae includes *Gobiobotia*, *XenophysogobioSaurogobio*, *Pseudogobio*, *Platysmacheilus*, *Biwia*, *Microphysogobio*, *Romanogobio*, *Abbottina*, *Acanthogobio*, *Gobio*, and *Ladislavia* (BS = 85%, PP = 97%). Within the group, *Ladislaviataczanowskii* was at the basal position. The phylogenetic tree from mtDNA supported *Ladislavia* should be included in the Gobioninae group (Tang et al. 2011). Bănărescu and Nalbant (1973) highlighted that *Acanthogobio* seemed to be a morphologically derived species of *Gobio*, as confirmed in our study. *Microphysogobio* is not monophyletic because of the placement of *Biwia*, *Romanogobio*, and *Platysmacheilus* which are found nested within *Microphysogobio*; this is in accordance with previous studies based on mitochondrial and nuclear genes (Yang et al. 2006; Tang et al. 2011). In morphology, *P.exiguous* and *Microphysogobio* showed similar characteristics that were a single row of dentition, with indicated that the evolutionary process was the decreasing number of teeth rows (Yu and Liu 2011). The taxonomic status of *Microphysogobio* remains uncertain because its putative member species were found to be broadly polyphyletic.

The tribe Sarcocheilichthyini includes *Coreius*, *Coreoleuciscus*, *Gnathopogon*, *Paracanthobrama*, *Gobiocypris*, *Pungtungia*, *Pseudopungtungia*, *Pseudorasbora*, *Rhinogobio*, and *Sarcocheilichthys* (BS = 86%, PP = 100%). Based on our trees, *Pungtungiaherzi* was assigned to *Pseudopungtungia*, and a grouping like this has been proposed in an earlier study (Kim et al. 2013). Our results and a previous study by Kim et al. (2013) suggested an unstable taxonomic status of the *Pseudopunungtungia* genus, which is polyphyletic. The placement of *Gobiocypris* within the *Gnathopogon* gives support to *Gobiocypris* as a subgenus of *Gnathopogon* (Tang et al. 2011). Moreover, we found that *Paraleucogobio* was also included in *Gnathopogon*, so we speculated that Paraleucogobio might also be a subgenus of Gnathopogon. Surprisingly, the phylogenetic tree showed that *Sarcocheilichthysbiwaensis* and *S.variegatusmicrooculus* had almost non-existent branch lengths. Komiya (2014) et al. suggested multiple colonization events of Lake Biwa by *S.biwaensis* and *S.v.microoculus* and confirmed the rapid speciation of *S.biwaensis* from an ancestral *S.v.microoculus* form. Therefore, we surmise that *S.biwaensis* and *S.v.microoculus* probably have mitochondrial introgression. Introgressive hybridization was not rare between closely related species (Yang et al. 2006).

Several monophyletic clades of *Coreius*, *Coreoleuciscus*, *Pseudorasbora*, *Rhinogobio*, *Sarcocheilichthys*, *Gobiobotia*, *Xenophysogobio*, *Saurogobio*, *Pseudogobio*, *Abbottina*, and *Squalidus* were supported (Fig. 2). The monophyletic of *Sarcocheilichthys*, *Rhinogobio*, *Coreius*, *Gobiobotia*, *Saurogobio*, *Pseudogobio*, and *Squalidus* are consistent with the phylogenetic results of Yang et al. (2006) on 44 species of Gobioninae using the mitochondrial *Cyt b*. Our results showed that phylogenetic analyses utilizing mitogenome sequences partially resolved genus- and species-level relationships within Gobioninae. However, extensive taxon sampling is required to completely resolve the relationships within the subfamily Gobioninae.

## Conclusions

In the present study, we sequenced and described the complete *M.elongatus* mitogenome (16,612 bp) that contains 37 genes and one control region as typical for vertebrate mitogenomes. The characteristics of the newly sequenced mitogenome are mostly consistent with those reported in other *Microphysogobio* mitogenomes. The subfamily Gobioninae was composed of three major lineages, and the phylogenetic trees strongly supported the non-monophyly of *Microphysogobio*. The results of the present study will be useful for further investigation of the evolutionary relationships within Gobioninae.

## Supplementary materials

Supplementary material 1 Table S1. Primers used for PCR Data type molecula data https://binary.pensoft.net/file/594675This dataset is made available under the Open Database License (http://opendatacommons.org/licenses/odbl/1.0/). The Open Database License (ODbL) is a license agreement intended to allow users to freely share, modify, and use this Dataset while maintaining this same freedom for others, provided that the original source and author(s) are credited.Renyi Zhang, Qian Tang, Lei Deng

Supplementary material 2 Table S2. List of species used to construct the phylogenetic tree in the present study Data type molecula data https://binary.pensoft.net/file/594676This dataset is made available under the Open Database License (http://opendatacommons.org/licenses/odbl/1.0/). The Open Database License (ODbL) is a license agreement intended to allow users to freely share, modify, and use this Dataset while maintaining this same freedom for others, provided that the original source and author(s) are credited.Renyi Zhang, Qian Tang, Lei Deng

Supplementary material 3 Table S3. Codon usage in the PCGs of the *Microphysogobioelongatus* mitogenome Data type molecula data https://binary.pensoft.net/file/594677This dataset is made available under the Open Database License (http://opendatacommons.org/licenses/odbl/1.0/). The Open Database License (ODbL) is a license agreement intended to allow users to freely share, modify, and use this Dataset while maintaining this same freedom for others, provided that the original source and author(s) are credited.Renyi Zhang, Qian Tang, Lei Deng

Supplementary material 4 Table S4. PartitionFinder results Data type molecula data https://binary.pensoft.net/file/594678This dataset is made available under the Open Database License (http://opendatacommons.org/licenses/odbl/1.0/). The Open Database License (ODbL) is a license agreement intended to allow users to freely share, modify, and use this Dataset while maintaining this same freedom for others, provided that the original source and author(s) are credited.Renyi Zhang, Qian Tang, Lei Deng

Supplementary material 5 Figure S1. Relative synonymous codon usage (RSCU) in the *M.elongatus* mitogenome Data type molecula data Explanation note Codon families are provided on the X-axis and the RSCU values on the Y-axis. https://binary.pensoft.net/file/594679This dataset is made available under the Open Database License (http://opendatacommons.org/licenses/odbl/1.0/). The Open Database License (ODbL) is a license agreement intended to allow users to freely share, modify, and use this Dataset while maintaining this same freedom for others, provided that the original source and author(s) are credited.Renyi Zhang, Qian Tang, Lei Deng

Supplementary material 6 Figure S2. Codon distribution in the *M.elongatus* mitogenome Data type molecula data Explanation note CDspT, codons per thousand codons. Codon families are provided on the X-axis. https://binary.pensoft.net/file/594680This dataset is made available under the Open Database License (http://opendatacommons.org/licenses/odbl/1.0/). The Open Database License (ODbL) is a license agreement intended to allow users to freely share, modify, and use this Dataset while maintaining this same freedom for others, provided that the original source and author(s) are credited.Renyi Zhang, Qian Tang, Lei Deng

Supplementary material 7 Figure S3. Putative secondary structures of the 22 tRNA genes identified in the mitochondrial genome of *M.elongatus* Data type molecula data Explanation note All tRNA genes are shown in the order of occurrence in the mitochondrial genome starting from tRNA^Phe^. The tRNAs are labelled with abbreviations of their corresponding amino acid. Dashed lines (-) indicate Watson-Crick base pairings. https://binary.pensoft.net/file/594681This dataset is made available under the Open Database License (http://opendatacommons.org/licenses/odbl/1.0/). The Open Database License (ODbL) is a license agreement intended to allow users to freely share, modify, and use this Dataset while maintaining this same freedom for others, provided that the original source and author(s) are credited.Renyi Zhang, Qian Tang, Lei Deng

Supplementary material 8 Figure S4. Control region of the *M.elongatus* mitochondrial genome Data type molecula data Explanation note The termination associated sequence domain (TAS), the central conserved domains (CSB-F, CSB-E, CSB-D) and the conserved sequence block domains (CSB-1, CSB-2, CSB-3) are shown in red font, and the conserved sequences are marked by black font and underlined. https://binary.pensoft.net/file/594682This dataset is made available under the Open Database License (http://opendatacommons.org/licenses/odbl/1.0/). The Open Database License (ODbL) is a license agreement intended to allow users to freely share, modify, and use this Dataset while maintaining this same freedom for others, provided that the original source and author(s) are credited.Renyi Zhang, Qian Tang, Lei Deng

Supplementary material 9 Figure S5. Phylogenetic relationships of Gobioninae based on complete mitochondrial genomes using Bayesian analyses Data type phylogenetic data Explanation note Bayesian posterior probabilities are shown at the nodes. https://binary.pensoft.net/file/594683This dataset is made available under the Open Database License (http://opendatacommons.org/licenses/odbl/1.0/). The Open Database License (ODbL) is a license agreement intended to allow users to freely share, modify, and use this Dataset while maintaining this same freedom for others, provided that the original source and author(s) are credited.Renyi Zhang, Qian Tang, Lei Deng

## References

[B1] Bănărescu P Nalbant TT (1973) Pisces, Teleostei, Cyprinidae (Gobioninae). Das Tierreich, Lieferung 93. Walter de Guryter, Berlin.

[B2] Bănărescu P (1992) A critical updated checklist of Gobioninae (Pisces, Cyprinidae). Travaux du Muséum d’Histoire Naturelle ‘‘Grigore Antipa’’ 32: 303–330.

[B3] Bernt M Donath A Jühling F Externbrink F Florentz C Fritzsch G Pütz J Middendorf M Stadler PF (2013) MITOS: improved *de novo* metazoan mitochondrial genome annotation. Molecular Phylogenetics and Evolution 69: 313–319. 10.1016/j.ympev.2012.08.023 22982435

[B4] Boore JL (1999) Animal mitochondrial genomes. Nucleic Acids Research 27: 1767–1780. 10.1093/nar/27.8.1767 10101183 PMC148383

[B5] Broughton RE Milam JE Roe BA (2001) The complete sequence of the zebrafish (*Danio rerio)* mitochondrial genome and evolutionary patterns in vertebrate mitochondrial DNA. Genome Research 11: 1958–1967. 10.1101/gr.156801 11691861 PMC311132

[B6] Cheng CY Wang JP Ho CW Cheng JW Ueng YT (2015) Complete mitochondrial DNA genome of *Microphysogobiobrevirostris* (Cypriniformes: Cyprinidae). Mitochondrial DNA 26: 293–294. 10.3109/19401736.2013.825781 24047164

[B7] He D Chen Y (2006) Biogeography and molecular phylogeny of the genus *Schizothorax* (Teleostei: Cyprinidae) in China inferred from cytochrome b sequences. Journal of Biogeography 33: 1448–1460. 10.1111/j.1365-2699.2006.01510.x

[B8] Huang SP Chen IS Shao KT (2016) A new species of *Microphysogobio* (Cypriniformes: Cyprinidae) from Fujian Province, China, and a molecular phylogenetic analysis of *Microphysogobio* species from southeastern China and Taiwan. Proceedings of the Biological Society of Washington 129: 195–211. 10.2988/0006-324X-129.Q3.195

[B9] Huang SP Zhao Y Chen IS Shao KT (2017) A new species of *Microphysogobio* (Cypriniformes: Cyprinidae) from Guangxi province, southern China. Zoological Studies 56: e8. 10.6620/ZS.2017.56-08 PMC6517699 31966207

[B10] Hwang DS Song HB Lee JS (2014) Complete mitochondrial genome of the freshwater gudgeon, *Microphysogobiokoreensis* (Cypriniformes, Cyprinidae). Mitochondrial DNA 25: 15–16. 10.3109/19401736.2013.775267 23488919

[B11] Imoto JM Saitoh K Sasaki T Yonezawa T Adachi J Kartavtsev YP Miya M Nishida M Hanzawa N (2013) Phylogeny and biogeography of highly diverged freshwater fish species (Leuciscinae, Cyprinidae, Teleostei) inferred from mitochondrial genome analysis. Gene 514: 112–124. 10.1016/j.gene.2012.10.019 23174367

[B12] Iwasaki W Fukunaga T Isagozawa R Yamada K Maeda Y Satoh TP Sado T Mabuchi K Takeshima H Miya M Nishida M (2013) MitoFish and MitoAnnotator: a mitochondrial genome database of fish with an accurate and automatic annotation pipeline. Molecular Biology and Evolution 30: 2531–2540. 10.1093/molbev/mst141 23955518 PMC3808866

[B13] Jiang ZG Gao EH Zhang E (2012) *Microphysogobionudiventris*, a new species of gudgeon (Teleostei: Cyprinidae) from the middle Chang-Jiang (Yangtze River) basin, Hubei Province, South China. Zootaxa 3586: 211–221. 10.11646/zootaxa.3586.1.19

[B14] Katoh K Standley DM (2013) MAFFT multiple sequence alignment software version 7: improvements in performance and usability. Molecular Biology and Evolution 30: 772–780. 10.1093/molbev/mst010 23329690 PMC3603318

[B15] Kim KY Lim YH Bang IC Nam YK (2009) Phylogenetic relationships among three new *Hemibarbus* mitogenome sequences belonging to the subfamily Gobioninae (Teleostei, Cypriniformes, and Cyprinidae). Mitochondrial DNA 20: 119–125. 10.3109/19401730903176896 19900061

[B16] Kim KY Ko MH Liu HZ Tang QY Chen XL Miyazaki JI Bang IC (2013) Phylogenetic relationships of *Pseudorasbora*, *Pseudopungtungia*, and *Pungtungia* (Teleostei; Cypriniformes; Gobioninae) inferred from multiple nuclear gene sequences. BioMed Research International 2013: 347242. 10.1155/2013/347242 PMC3782758 24106702

[B17] Komiya T Yanagibayashi SF Watanabe K (2014) Multiple colonizations of Lake Biwa by *Sarcocheilichthys* fishes and their population history. Environmental Biology of Fishes 97(7): 741–755. 10.1007/s10641-013-0176-9

[B18] Lalitha S (2000) Primer Premier 5. Biotech Software & Internet Report 1: 270–272. 10.1089/152791600459894

[B19] Lanfear R Frandsen PB Wright AM Senfeld T Calcott B (2017) PartitionFinder 2: new methods for selecting partitioned models of evolution for molecular and morphological phylogenetic analyses. Molecular Biology and Evolution 34: 772–773. 10.1093/molbev/msw260 28013191

[B20] Li J Li XH Jia XP Tan XC Wang C Li YF Shao XF (2012) Relationship between fish community diversity and environmental factors in the Lianjiang River, Guangdong, China. Acta Ecologica Sinica 32: 5795–5805. 10.5846/stxb201108041142

[B21] Lin DY Lin HD Tzeng SJ Chiang TY (2014) Complete mitochondrial genome of *Microphysogobioalticorpus* (Cypriniformes, Cyprinidae). Mitochondrial DNA 25: 173–174. 10.3109/19401736.2013.792062 23631369

[B22] Liu H Yang J Tang Q (2010) Estimated evolutionary tempo of East Asian gobionid fishes (Teleostei: Cyprinidae) from mitochondrial DNA sequence data. Chinese Science Bulletin 55: 1501–1510. 10.1007/s11434-010-3159-7

[B23] Liu Y Hou XF Zhou J (2013) Fish species composition in Shibing of Guizhou, Southwest China, a candidate World Heritage Site. Chinese Journal of Applied Ecology 32: 1850–1856. 10.13292/j.1000-4890.2013.0394

[B24] Lowe TM Chan PP (2016) tRNAscan-SE On-line: integrating search and context for analysis of transfer RNA genes. Nucleic Acids Research 44: W54–W57. 10.1093/nar/gkw413 PMC4987944 27174935

[B25] Minh BQ Nguyen MAT von Haeseler A (2013) Ultrafast approximation for phylogenetic bootstrap. Molecular Biology and Evolution 30: 1188–1195. 10.1093/molbev/mst024 23418397 PMC3670741

[B26] Miya M Nishida M (2000) Use of mitogenomic information in teleostean molecular phylogenetics: a tree-based exploration under the maximum-parsimony optimality criterion. Molecular Phylogenetics and Evolution 17: 437–455. 10.1006/mpev.2000.0839 11133198

[B27] Miya M Takeshima H Endo H Ishiguro NB Inoue JG Mukai T Satoh TP Yamaguchi M Kawaguchi A Mabuchi K (2003) Major patterns of higher teleostean phylogenies: a new perspective based on 100 complete mitochondrial DNA sequences. Molecular Phylogenetics and Evolution 26: 121–138. 10.3897/10.1016/S1055-7903(02)00332-9 12470944

[B28] Nguyen LT Schmidt HA Haeseler AV Minh BQ (2015) IQ-TREE: a fast and effective stochastic algorithm for estimating maximum-likelihood phylogenies. Molecular Biology and Evolution 32: 268–274. 10.1093/molbev/msu300 25371430 PMC4271533

[B29] Ojala D Montoya J Attardi G (1981) tRNA punctuation model of RNA processing in human mitochondria. Nature 290: 470–474. 10.1038/290470a0 7219536

[B30] Perna NT Kocher TD (1995) Patterns of nucleotide composition at fourfold degenerate sites of animal mitochondrial genomes. Journal of Molecular Evolution 41: 353–358. 10.1007/BF00186547 7563121

[B31] Ronquist F Teslenko M van der Mark P Ayres Daniel L Darling A Höhna S Larget B Liu L Suchard MA Huelsenbeck JP (2012) MrBayes 3.2: efficient Bayesian phylogenetic inference and model choice across a large model space. Systematic Biology 61: 539–542. 10.1093/sysbio/sys029 22357727 PMC3329765

[B32] Sambrook J Fritsch EF Maniatis T (1989) Molecular Cloning: a Laboratory Manual. 2^nd^ edn. Cold Spring Harbor Press, New York, 1695 pp.

[B33] Saitoh K Sado T Mayden RL Hanzawa N Nakamura K Nishida M Miya M (2006) Mitogenomic evolution and interrelationships of the Cypriniformes (Actinopterygii: Ostariophysi): the first evidence toward resolution of higher-level relationships of the world’s largest freshwater fish clade based on 59 whole mitogenome sequences. Journal of Molecular Evolution 63: 826–884. 10.1007/s00239-005-0293-y 17086453

[B34] Sharma A Siva C Ali S Sahoo PK Nath R Laskar MA Sarma D (2020) The complete mitochondrial genome of the medicinal fish, *Cyprinionsemiplotum*: Insight into its structural features and phylogenetic implications. International Journal of Biological Macromolecules 164: 939–948. 10.1016/j.ijbiomac.2020.07.142 32687902

[B35] Sun ZX Kawase S Zhang R Zhao YH (2021) Taxonomic revision and redescription of *Microphysogobiohsinglungshanensis*, the type species of *Microphysogobio* Mori, 1934 (Cypriniformes: Cyprinidae). Journal of Fish Biology 2021: 1–11. 10.1111/jfb.14725 33715166

[B36] Tamura K Stecher G Peterson D Filipski A Kumar S (2013) MEGA6: molecular evolutionary genetics analysis version 6.0. Molecular Phylogenetics and Evolution 30: 2725–2729. 10.1093/molbev/mst197 PMC3840312 24132122

[B37] Tang KL Agnew MK Chen WJ Hirt MV Raley ME Sado T Schneider LM Yang L Bart HL He S Liu H Miya M Saitoh K Simons AM Wood RM Mayden RL (2011) Phylogeny of the gudgeons (Teleostei: Cyprinidae: Gobioninae). Molecular Phylogenetics and Evolution 61: 103–124. 10.1016/j.ympev.2011.05.022 21672635

[B38] Wang IC Lin HD Liang CM Huang CC Wang RD Yang JQ Wang WK (2020) Complete mitochondrial genome of the freshwater fish *Onychostomalepturum* (Teleostei, Cyprinidae): genome characterization and phylogenetic analysis. ZooKeys 1005: 57–72. 10.3897/zookeys.1005.57592 33390755 PMC7765746

[B39] Wang X (2019) Construction of fish DNA barcode database and excavation of cryptical species in Henan Province. Master thesis, Henan Normal University, Xinxiang.

[B40] Wang W He S Chen Y (2002) Mitochondrial d-loop sequence variation and phylogeny of gobiobotine fishes. Progress in Natural Science 12: 866–868.

[B41] Wolstenholme DR (1992) Animal mitochondrial DNA: structure and evolution. International Review of Cytology 141: 173–216. 10.1016/S0074-7696(08)62066-5 1452431

[B42] Xiao WH Zhang YP (2000) Genetics and evolution of mitochondrial DNA in fish. Acta Hydrobiologica Sinica 24: 384–391. 10.3321/j.issn:1000-3207.2000.04.014

[B43] Yamanoue Y Miya M Matsuura K Yagishita N Mabuchi K Sakai H Katoh M Nishida M (2007) Phylogenetic position of tetraodontiform fishes within the higher teleosts: Bayesian inferences based on 44 whole mitochondrial genome sequences. Molecular Phylogenetics and Evolution 45: 89–101. 10.1016/j.ympev.2007.03.008 17490896

[B44] Yang J He S Freyhof J Witte K Liu H (2006) The phylogenetic relationships of the Gobioninae (Teleostei: Cyprinidae) inferred from mitochondrial cytochrome b gene sequences. Hydrobiologia 553: 255–266. 10.1007/s10750-005-1301-3

[B45] Yu Z Liu H (2011) The evolution of pharyngeal bones and teeth in Gobioninae fishes (Teleostei: Cyprinidae) analyzed with phylogenetic comparative methods. Hydrobiologia 664: 183–197. 10.1007/s10750-010-0598-8

[B46] Yue PQ (1998) Gobioninae. In: Chen YY (Ed.) Fauna Sinica, Osteichthyes, Cypriniformes (II).Science Press, Beijing, 232–378.

[B47] Zhang D Gao F Jakovlić I Zou H Zhang J Li WX Wang GT (2020) PhyloSuite: an integrated and scalable desktop platform for streamlined molecular sequence data management and evolutionary phylogenetics studies. Molecular Ecology Resources 20: 348–355. 10.1111/1755-0998.13096 31599058

[B48] Zhang F Wu HH Yang CX Li CL Wang YF Gao YN Gu QH Zhou CJ Meng XL Nie GX (2018) Length-weight relationships of 5 Gobioninae species from the Yellow River basin and Huaihe River basin in Henan Province, China. Journal of Applied Ichthyology 34: 1320–1323. 10.1111/jai.13793

[B49] Zhang R Wang X (2018) Characterization and phylogenetic analysis of the complete mitogenome of a rare cavefish, *Sinocyclocheilusmultipunctatus* (Cypriniformes: Cyprinidae). Genes & Genomics 40: 1033–1040. 10.1007/s13258-018-0711-3 29949074

[B50] Zhao J Xu D Zhao K Diogo R Yang J Peng Z (2016) The origin and divergence of Gobioninae fishes (Teleostei: Cyprinidae) based on complete mitochondrial genome sequences. Journal of Applied Ichthyology 32: 32–39. 10.1111/jai.12920

[B51] Zhong L Wang M Li D Tang S Zhang T Bian W Chen X (2018) Complete mitochondrial genome of *Odontobutishaifengensis* (Perciformes, Odontobutiae): a unique rearrangement of tRNAs and additional non-coding regions identified in the genus *Odontobutis*. Genomics 110: 382–388. 10.1016/j.ygeno.2017.12.008 29262306

