# Supplementary material for: The complete mitochondrial genome of Microphysogobioelongatus (Teleostei, Cyprinidae) and its phylogenetic implications
Source: Zookeys. 2021 Oct 1;1061:57–73. doi: 10.3897/zookeys.1061.70176 (PMC8501002; doi:10.3897/zookeys.1061.70176)
Supplement: Supplementary material 1 — Table S1. Primers used for PCR [file zookeys-1061-057-s001.docx]

**Table S1** Primers used for PCR.

| **Primer name** | **Primer sequence** | **Annealing T (**°C**)** |
| --- | --- | --- |
| MF1 | 5′-GGACAAGGAGCGGGCATC-3′ | 55 |
| MR1 | 5′-CGGGTTTTGGTCTGGGGT-3′ |  |
| MF2 | 5′-CGCAAGGGAACGCTGAAAG-3′ | 53 |
| MR2 | 5′-GCCCCAACCGAAGGTAGAA-3′ |  |
| MF3 | 5′-ATGAATGGCTAAACGAGG-3′ | 45 |
| MR3 | 5′-ATTGGGGAAGTGAGAAGA-3′ |  |
| MF4 | 5′-ACGTAGAATACGCGGGGGGA-3′ | 51 |
| MR4 | 5′-TTGTGGTTGAAATGGTTAGG-3′ |  |
| MF5 | 5′-CCGCCCTTGTGTTATTATCA-3′ | 48 |
| MR5 | 5′-GCACGGGTGTCTACATCTAT-3′ |  |
| MF6 | 5′-GCCTACTACGCTGGTAAAAA-3′ | 47 |
| MR6 | 5′-ATGGGAGAGCAATCAAAACT-3′ |  |
| MF7 | 5′-GGCACATCCCACACAACT-3′ | 44 |
| MR7 | 5′-TGGTACGGCAAGTCCTAT-3′ |  |
| MF8 | 5′-AACCGACTTATTACCCTCC-3′ | 46 |
| MR8 | 5′-CTGTTTTCGTTCTCCCTCT-3′ |  |
| MF9 | 5′-TTCCTCGGATTTTTCTGA-3′ | 45 |
| MR9 | 5′-TAGGCTGTGGGTGATTGT-3′ |  |
| MF10 | 5′-GCTCCTATGCTCCTACTA-3′ | 43 |
| MR10 | 5′-TTGATTTCTGTCCTTCCT-3′ |  |
| MF11 | 5′-AAACCAGAACTAATGTGAGG-3′ | 46 |
| MR11 | 5′-GATAAGTGTAAGGACGAGGG-3′ |  |
| MF12 | 5′-TTGAAGCCCTAAACACCTC-3′ | 48 |
| MR12 | 5′-ACTGTAGCCCCTCAGAAAG-3′ |  |
| MF13 | 5′-CTATAAAGAAACCTGAAATATCG-3′ | 43 |
| MR13 | 5′-AAGGTAACCCAAAAAGGAGAA-3′ |  |
| MF14 | 5′-TAATGATAGAATCAGGGACAC-3′ | 42 |
| MR14 | 5′-GCTTACTTTTATTACCTTCAC-3′ |  |
