# Supplementary material for: The complete mitochondrial genome of Microphysogobioelongatus (Teleostei, Cyprinidae) and its phylogenetic implications
Source: Zookeys. 2021 Oct 1;1061:57–73. doi: 10.3897/zookeys.1061.70176 (PMC8501002; doi:10.3897/zookeys.1061.70176)
Supplement: Supplementary material 3 — Table S3. Codon usage in the PCGs of the Microphysogobioelongatus mitogenome [file zookeys-1061-057-s003.docx]

**Table S3** Codon usage in the PCGs of the *Microphysogobio elongatus* mitogenome.

| **Codon(AA)** | **Count** | **RSCU** | **Codon(AA)** | **Count** | **RSCU** | **Codon(AA)** | **Count** | **RSCU** | **Codon(AA)** | **Count** | **RSCU** |
| --- | --- | --- | --- | --- | --- | --- | --- | --- | --- | --- | --- |
| UUU(F) | 82 | 0.94 | UCU(S) | 76 | 1.38 | UAU(Y) | 77 | 0.96 | UGU(C) | 25 | 0.91 |
| UUC(F) | 93 | 1.06 | UCC(S) | 72 | 1.31 | UAC(Y) | 83 | 1.04 | UGC(C) | 30 | 1.09 |
| UUA(L) | 117 | 1.28 | UCA(S) | 89 | 1.62 | UAA(*) | 68 | 0.96 | UGA(*) | 86 | 1.21 |
| UUG(L) | 59 | 0.64 | UCG(S) | 24 | 0.44 | UAG(*) | 59 | 0.83 | UGG(W) | 27 | 1 |
| CUU(L) | 90 | 0.98 | CCU(P) | 55 | 0.85 | CAU(H) | 60 | 0.82 | CGU(R) | 11 | 0.53 |
| CUC(L) | 73 | 0.8 | CCC(P) | 85 | 1.31 | CAC(H) | 87 | 1.18 | CGC(R) | 14 | 0.68 |
| CUA(L) | 159 | 1.74 | CCA(P) | 95 | 1.46 | CAA(Q) | 83 | 1.3 | CGA(R) | 39 | 1.89 |
| CUG(L) | 51 | 0.56 | CCG(P) | 25 | 0.38 | CAG(Q) | 45 | 0.7 | CGG(R) | 25 | 1.21 |
| AUU(I) | 143 | 1.39 | ACU(T) | 69 | 0.91 | AAU(N) | 60 | 0.92 | AGU(S) | 28 | 0.51 |
| AUC(I) | 59 | 0.57 | ACC(T) | 97 | 1.28 | AAC(N) | 71 | 1.08 | AGC(S) | 41 | 0.75 |
| AUA(I) | 106 | 1.03 | ACA(T) | 112 | 1.47 | AAA(K) | 67 | 1.47 | AGA(R) | 17 | 0.82 |
| AUG(M) | 61 | 1 | ACG(T) | 26 | 0.34 | AAG(K) | 24 | 0.53 | AGG(R) | 18 | 0.87 |
| GUU(V) | 29 | 0.78 | GCU(A) | 32 | 0.56 | GAU(D) | 39 | 0.93 | GGU(G) | 32 | 0.71 |
| GUC(V) | 27 | 0.72 | GCC(A) | 107 | 1.86 | GAC(D) | 45 | 1.07 | GGC(G) | 38 | 0.84 |
| GUA(V) | 64 | 1.72 | GCA(A) | 82 | 1.43 | GAA(E) | 69 | 1.35 | GGA(G) | 61 | 1.36 |
| GUG(V) | 29 | 0.78 | GCG(A) | 9 | 0.16 | GAG(E) | 33 | 0.65 | GGG(G) | 49 | 1.09 |

Notes: A total of 3808 codons for *M. elongatus* were analyzed. AA, amino acid; RSCU = relative synonymous codon usage, *, Stop codon.
