# Supplementary material for: The complete mitochondrial genome of Microphysogobioelongatus (Teleostei, Cyprinidae) and its phylogenetic implications
Source: Zookeys. 2021 Oct 1;1061:57–73. doi: 10.3897/zookeys.1061.70176 (PMC8501002; doi:10.3897/zookeys.1061.70176)
Supplement: Supplementary material 4 — Table S4. PartitionFinder results [file zookeys-1061-057-s004.docx]

**Table S4** PartitionFinder Results.

| Subset | Partition | Model |
| --- | --- | --- |
| 1 | atp6_codon1 | GTR+I+G |
| 2 | nad4_codon2, nad3_codon2, atp6_codon2 | GTR+I+G |
| 3 | atp6_codon3 | GTR+I+G |
| 4 | atp8_codon1, nad2_codon1 | GTR+I+G |
| 5 | nad5_codon2, atp8_codon2 | GTR+I+G |
| 6 | cox2_codon3, atp8_codon3 | GTR+I+G |
| 7 | cox1_codon1 | GTR+I+G |
| 8 | cox1_codon2, cox3_codon2 | K81UF+I+G |
| 9 | cox1_codon3 | GTR+I+G |
| 10 | cox2_codon1 | TRN+I+G |
| 11 | cox2_codon2, nad4L_codon2 | TVM+I |
| 12 | cox3_codon1 | K81+I+G |
| 13 | cox3_codon3, nad4L_codon3, nad3_codon3 | GTR+I+G |
| 14 | cytb_codon1, nad1_codon1 | GTR+I+G |
| 15 | cytb_codon2 | TRN+I+G |
| 16 | cytb_codon3 | GTR+I+G |
| 17 | nad1_codon2 | GTR+I+G |
| 18 | nad1_codon3, nad2_codon3 | GTR+I+G |
| 19 | nad2_codon2 | GTR+I+G |
| 20 | nad3_codon1, nad4L_codon1 | GTR+I+G |
| 21 | nad4_codon1 | GTR+I+G |
| 22 | nad4_codon3 | GTR+I+G |
| 23 | nad5_codon1 | GTR+I+G |
| 24 | nad5_codon3 | GTR+I+G |
| 25 | nad6_codon1 | GTR+I+G |
| 26 | nad6_codon2 | GTR+I+G |
| 27 | nad6_codon3 | GTR+I+G |
