# Supplementary figures and images for: The complete mitochondrial genome of Microphysogobioelongatus (Teleostei, Cyprinidae) and its phylogenetic implications
Source: Zookeys. 2021 Oct 1;1061:57–73. doi: 10.3897/zookeys.1061.70176 (PMC8501002; doi:10.3897/zookeys.1061.70176)

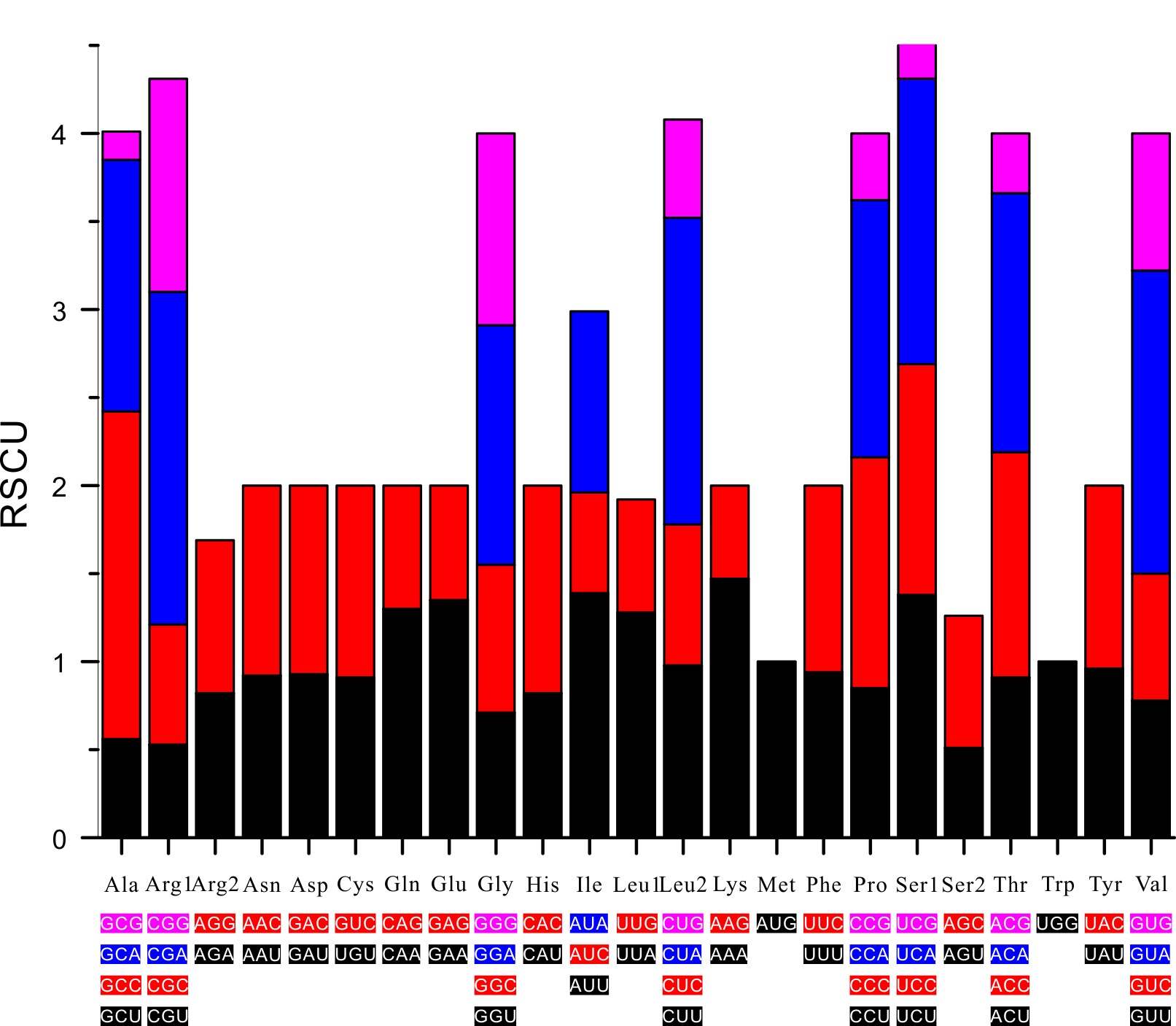

Supplement: Supplementary material 5 — Figure S1. Relative synonymous codon usage (RSCU) in the M.elongatus mitogenome [file zookeys-1061-057-s005.tif]

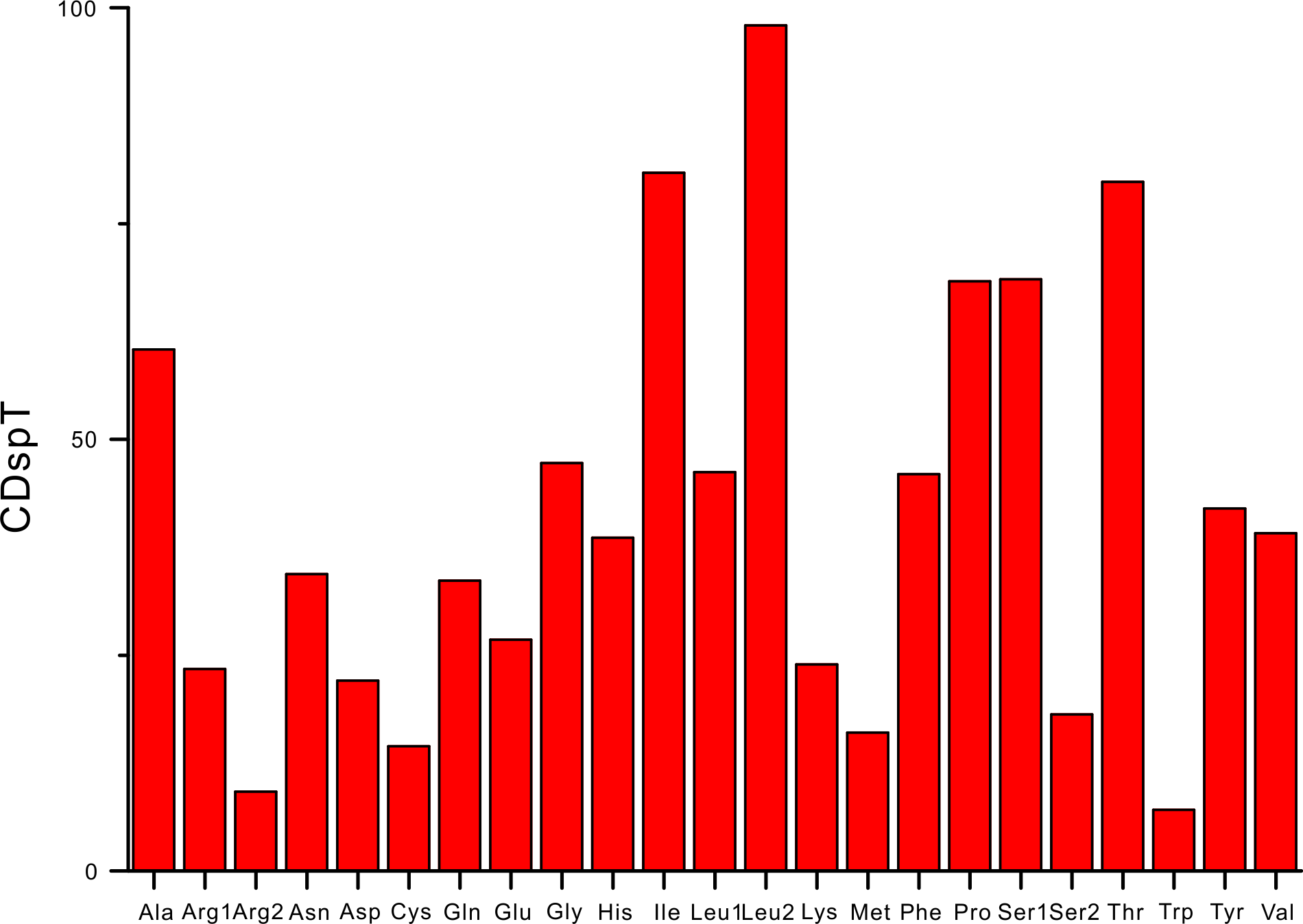

Supplement: Supplementary material 6 — Figure S2. Codon distribution in the M.elongatus mitogenome [file zookeys-1061-057-s006.tif]

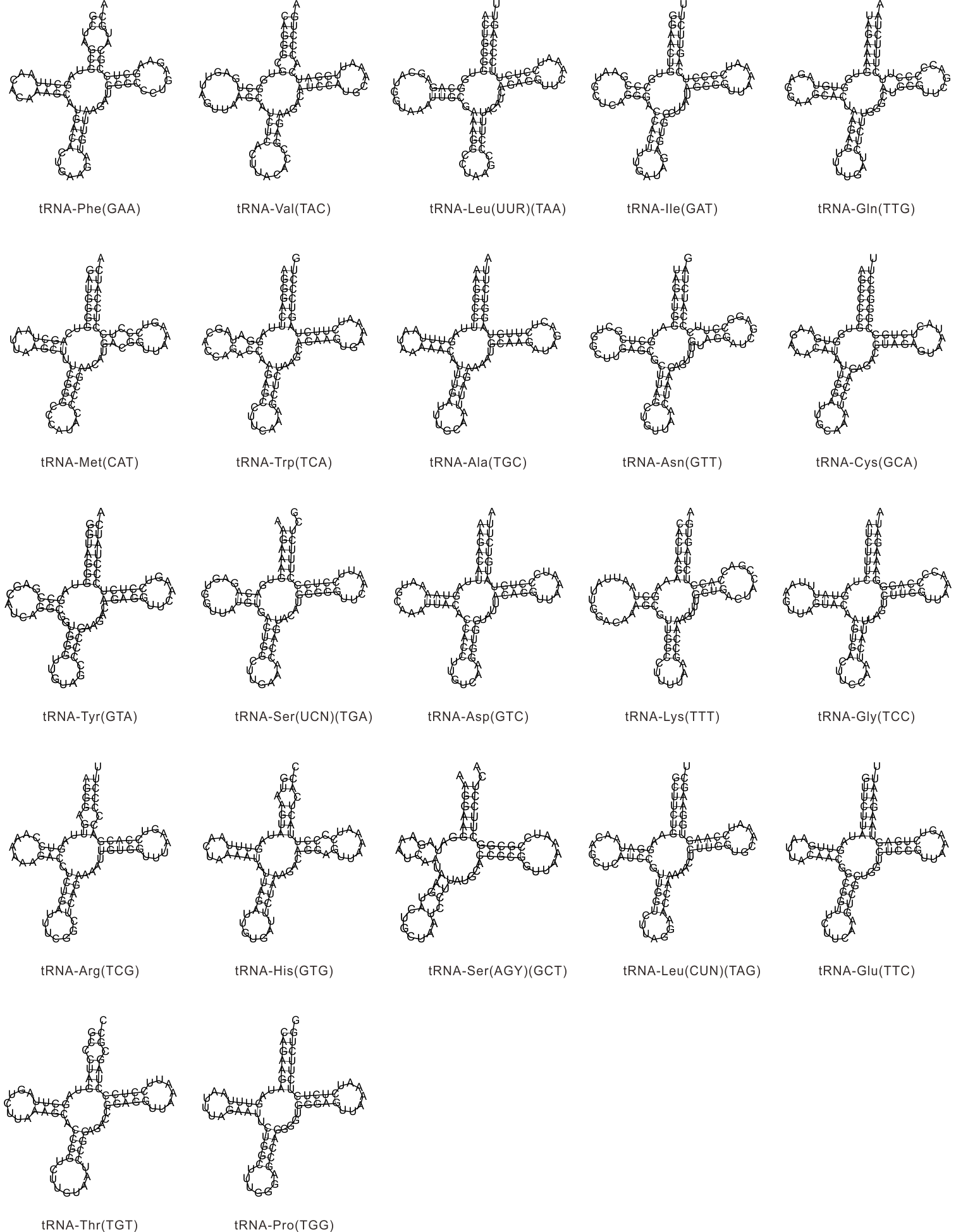

Supplement: Supplementary material 7 — Figure S3. Putative secondary structures of the 22 tRNA genes identified in the mitochondrial genome of M.elongatus [file zookeys-1061-057-s007.tif]

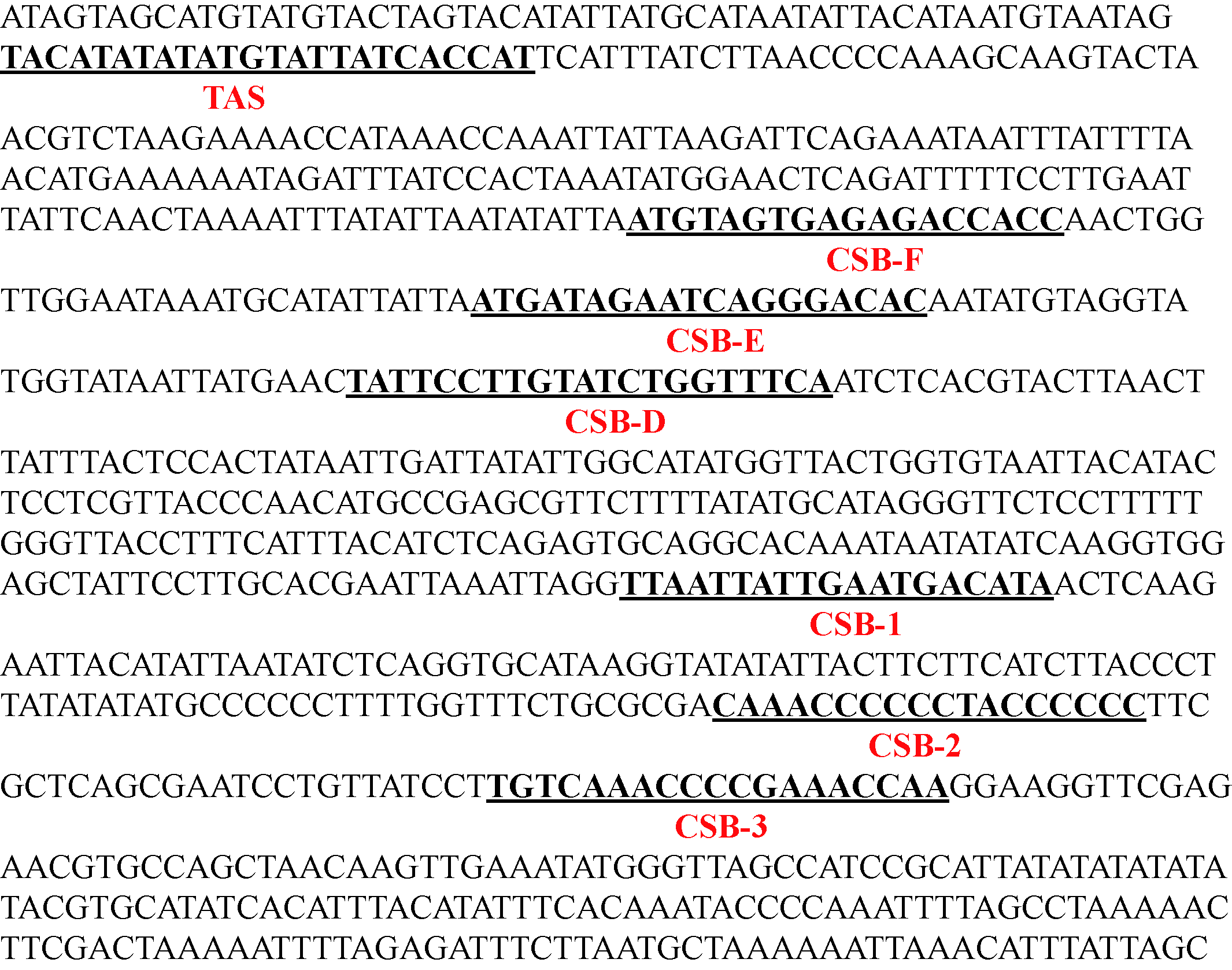

Supplement: Supplementary material 8 — Figure S4. Control region of the M.elongatus mitochondrial genome [file zookeys-1061-057-s008.tif]
